# Smallholders participation in sustainable certification: The mediating impact of deliberative communication and responsible leadership

**DOI:** 10.3389/fpsyg.2022.978993

**Published:** 2022-09-29

**Authors:** Ammar Redza Ahmad Rizal, Shahrina Md Nordin

**Affiliations:** ^1^Centre for Research in Media and Communication, Faculty of Social Sciences and Humanities, Universiti Kebangsaan Malaysia, Bangi, Malaysia; ^2^Institute of Sustainable Building, Universiti Teknologi PETRONAS, Seri Iskandar, Malaysia

**Keywords:** social structure, communication, leadership oil-palm, sustainable farming, farmers, green psychology

## Abstract

The initiative to ensure oil-palm smallholders around the world participate in sustainable certification is increasing. Different efforts were strategised including increasing awareness and providing financial support. Despite that, the number of smallholders’ participation in sustainable certification is relatively low. This study embarked on the objective to identify the role of social structure, namely social interaction ties in affecting smallholders’ participative behaviours. Moreover, this study is also looking on the mediating impact of deliberative communication and responsible leadership in explaining the relationship between the two previously stated constructs. Using a quantitative research design, this study collected data from 440 smallholders as its respondents. Samples were randomly selected, and questionnaires were distributed to obtain their responses. Data collected were then analysed using PLS-SEM to test the developed hypothesis. Accordingly, the findings indicate that social interaction ties have a significant impact on smallholders’ decisions to participate in sustainable certification. Furthermore, both deliberative communication and responsible leadership were proven to be significant mediators. This study provides insights on how smallholders’ participation in sustainable certification can be improved by tapping on the social structure elements as well as adopting deliberative communication and responsible leadership as a method to communicate and lead with the smallholders. This shall expand literature related to organisation psychology in rural areas and sustainability.

## Introduction

Sustainability certification has increasingly become a critical part of governance in the palm oil industry, especially in these recent years. Numerous efforts and initiatives have been spearheaded to undertake the challenges in addressing the issues raised by the public and Non-governmental Organisations. The industry is under immense pressure and scrutiny as the key players face criticisms, especially on environmental issues. In advocating and observing sustainability, the Roundtable of Sustainable Palm Oil (RSPO) was established in 2004 to offer a platform for a voluntary act in obtaining sustainable certification. Various other similar initiatives and standards have also been established for instance the Indonesian Sustainable Palm Oil (ISPO) and Malaysian Sustainable Palm Oil (MSPO) certifications which are mandatory to producers in each level of palm oil production (Abdul Aziz and Kuntom, 2016; Shahida et al., 2019). Despite the sustainable certification initiatives, only 30% of smallholders in Malaysia have sought certification with either MSPO or RSPO.

Certification for sustainability is essential for smallholders due to several reasons. Firstly, there would be a serious threat of disruption in the palm oil supply if the global. The market only allows for palm oil products with sustainable certifications in the global value chain. Export of palm oil products to European Union (EU) markets for example has already placed a restrain on products that are considered unsustainable (Kadarusman and Pramudya, 2019), due to the pledge made by the EU countries in the Renewable Energy Directive Council (Council of the European Union, 2018). The move also echoed in the resolution of the EU’s Parliament to ban palm oil by 2030 (European Parliament, 2018). This poses threats to Asian exporters like Malaysia and Indonesia (Kadarusman and Pramudya, 2019) especially when there is a huge number of smallholders who refuse to attain sustainability certifications in the country. The implication of this will affect the economy of smallholders where they would not be able to sell their products to the European market which is valued at USD 19.5 billion in 2022 and expected to grow to USD 27 billion by 2027 (Market Data Forecast, 2022). Such a huge loss will be detrimental to the livelihood of the smallholders.

Approximately 40–50% of oil palm plantations around the world are managed by smallholders with less than 5 hectares of land each (Jelsma et al., 2017; Hafizuddin-Syah et al., 2018). The rising global trade flow over the past decades provides opportunities for smallholders to participate and benefit from more commercialized global value chains (Rigg et al., 2016). Although the occasions lead to the possibility of an increase in profit and productivity amongst the smallholders, several concerns arise. First, the shift of power from farmers to processors/retailers due to the proliferation of safety and quality standards within the market create barriers to the participation of smallholders (Lee et al., 2012) that have also reduced their bargaining power. Moreover, the previous approach by institutions and government agencies that treat smallholders as a homogenous population during policy and standards development fails to adequately account for the wide range of issues faced (Jelsma et al., 2017).

From the perspective of organizational and institutional structure, smallholders form their cluster comprising several oil-palm smallholders. Together they share some of the essential resources for oil palm plantation such as knowledge, machinery and fruit dealers (Ador et al., 2016). Some even created a formal organisation in the form of cooperatives where more resources such as capital and labour are pooled together. Furthermore, revenue was shared amongst the members of organisations. Hence, it is not surprising that other institutions within the industry treat them as an organisation. For instance, in Malaysia, the authorised organisation to develop and advance the oil-palm industry – Malaysia Palm Oil Board (MPOB) created Sustainable Palm Oil Cluster (SPOC) to drive and hasten the adoption of sustainable certification by the smallholders (Ahmad Rizal et al., 2021).

Therefore, several steps were taken that mostly were aimed at increasing smallholders’ awareness through extension services and training. They also provided financial support to participate in the sustainable certification scheme (Aziz et al., 2021; Majid et al., 2021). With the financial incentive and technical support provided to the smallholders in the palm oil industry, it was expected that there would be a huge number of smallholders participating in sustainable certifications. It is in accordance with the perspective of game theory and rational choice theory where an individual shall maximize their own utility when given chance to act so (Grandori, 2010; Wong, 2014; Ahmad Rizal et al., 2021). Empirical evidence however proves otherwise. However, the effort has very minimal impact on sustainability in the Malaysian palm oil industry. A study indicates that the overall sustainability score for a typical crude palm oil supply chain in Malaysia is 3.47/5, which is below the sustainability target of 5/5 (Lim and Biswas, 2019). Furthermore, approximately only 30% of the independent smallholder’s plantations or about 331,740 hectares out of 986,331 hectares have obtained certification (MPOCC, 2018).

The underperformance of this initiative demands a better perspective in explaining smallholders’ behaviours in adopting a sustainable certification scheme. Several studies that are looking into farmers and organisations have been suggesting other factors to explain their behaviours in adopting innovation or initiative. Nordin and colleagues in their studies (Md Nordin et al., 2021) showed that farmers sometimes look into their peer practices and observe them before making any decision. According to Bandura, human beings look into other members of society to obtain new knowledge (Bandura, 2001). Similarly, Ajzen stated that acting by society’s norms affects one behaviour and attitude (Ajzen, 1991, 2020). In the organisational study, the process of sharing knowledge between members of an organization resulted in a “collective mind” which is pivotal for creating High- Reliability Organisation (HRO) (Haslam et al., 2022). In the context of this study, attention should be given to social structure where the factor could enhance the formation of collective minds which contributed to smallholders’ decision to participate in sustainable certification.

The social structure itself has proven to be essential to explain individual behaviours are affected by the group’s identity and norms. Studies reported that the social structure mechanism of function is affected by the production of social capital within the groups (Falk and Kilpatrick, 2000; Labianca et al., 2004). The capital comprising of trust, cooperation and coordination is produced when an individual interacts with the other individual and the constant exchange was argued to have an effect on individual behaviours (Davis, 2014). However, the current study of sustainability behaviours and rural society is not able to explain in detail the working mechanisms of social interaction. Thus, there is a need to move beyond and explore factors that explain the mechanism of social interaction.

Researchers argue that communication and leadership are among two important elements which define the society relationship (Putnam, 1995; Gutmann, 2009; Ahmad Rizal et al., 2022). It is argued that interaction and identity development within society can only be made possible with the existence of communication and leadership. For instance, lack of communication between identity groups is proven detrimental especially when there are cultural barriers between them (Gutmann, 2009, 68). Other studies indicate that leaders could improve coordination despite the relation being separated by structural holes (detrimental factor in social structure) (Lin et al., 2001, 49). On a similar ground, these findings necessitate further probing on whether social structure elements and their impact on smallholders’ participation behaviour could be explained by communication and leadership in the context of sustainability behaviour for individuals who are living and working in a group. This study looks into smallholders’ participative behaviour in a sustainable scheme to explain that phenomenon.

Thus, the main objective of this study is to investigate the impact of social structure through social interaction and the mediating impact of deliberative communication and responsible leadership on smallholders’ participative behaviour in sustainable certification schemes. This paper will first introduce the background of the study and followed it with a critical review of the literature. The paper will then discuss the hypothesis development, research methodology, findings and followed by a discussion and the conclusion.

The findings from this article will fill the gap in the current literature related to sustainability behaviour, and group psychology in the rural area. It will identify whether communication and leadership are proven to be factors in explaining the effect of social structure on sustainability behaviour. These findings will help the policymakers to develop a better strategic policy to increase smallholders’ participation in the sustainability scheme. Moreover, it will also help the policymakers and interested groups to further understand the mechanism of sustainable behaviour amongst groups and individuals who are living in rural area.

## Literature review

### Overcoming rational choice theory limitation

The current literature on sustainable certification largely emphasizes rational-choice theory (RCT) to explain the lack of participation by smallholders in the schemes (Hidayat et al., 2016; Ni et al., 2016; Ahmad Rizal et al., 2021), which has its limitation. RCT primarily focuses on human behaviour in optimizing economic values and utility (i.e., personal gain or loss based on self-interest). Such assumption is often overgeneralized when explaining human actions (Hodgson, 2012). RCT is criticised for “*its excessive quest for generality, it will fail to focus on the historically and geographically specific features of the socio-economic systems that we wish to study and understand*” (Hodgson, 2012, 104). The limitation of RCT calls for consideration of another perspective to explain the low participation of smallholder farmers in sustainability schemes.

Several approaches are used to attract smallholders’ participation for instance a grant for any smallholders to join the initiatives by MSPO (The Star, 2017), financial assistance certification cost (RSPO) and access to sell plantation output (i.e., Fresh Fruit Bunch, FFB) at a premium price (Johnson, 2014). These perks not only could reduce the costs but also increase the gain. Past studies argue that without such assistance, smallholders would not choose to participate in sustainability initiatives (Hidayat et al., 2016; Rietberg and Slingerland, 2016). Participation amongst smallholders is still low (MPOCC, 2018). This indicates that considerations based on RCT alone are insufficient.

By adhering to RCT, scholars and policymakers missed out on the important aspect of smallholders’ livelihood that encompasses social influence and social norms. RCT puts a large focus on human behaviour based on maximizing utility (i.e., personal gain based on self-interest). Most of the empirical evidence found will always correlate with the idea of RCT, making RCT looks like a universal theory of human action (Hodgson, 2012). However, RCT lacks explanation capability and does not consider important elements such as historical, cultural and institutional specificities of particular societies in his account (Hodgson, 2012). It is therefore why economists such as Hodgson argued that RCT problems fall on “*its excessive quest for generality, it will fail to focus on the historically and geographically specific features of the socio-economic systems that we wish to study and understand*” (Hodgson, 2012, 104).

Smallholders as important stakeholders where their knowledge and practices are deeply rooted within the social culture and structures. Understanding their social structure and system is hence of paramount importance in developing a framework for heightened participation in sustainable initiatives.

### Social structure as significant determinants

Smallholders, like family farmers, share a rural community social structure. Several families cluster together to form a community (Thompson, 2004). They live in a nucleus or extended family. Typically, the relationships between smallholders within a village are lateral, with informal inter-personal networks forming among the smallholders themselves (Rogers, 2003). The majority of their interactions take place in public settings, such as coffee shops, congregation halls, and community halls. A farmer’s ability to exhibit “good farming” methods to their peers determines how they are valued (Taylor and Van Grieken, 2015). In addition, they share information with other members of the group, including developments in technology and inventions to boost agricultural output and productivity (Nordin et al., 2015; Mannan et al., 2017; Ahmad Rizal et al., 2021).

Social interaction ties are the primary component of social structure. It illustrates the amount to which community members interact within the society. Interaction has been shown to be a crucial characteristic of effective social development. Adoption of innovation and implementation of the policies are correlated with strong relationships and interdependence between individual and other group members in society (Jenkins, 2014; Thévenot, 2014). Through social ties and interactions, the relationships form tight-knit cliques that are interconnected with other cliques. Individuals inside a clique are therefore linked to other cliques *via* weak relationships rather than strong ties. Nonetheless, “the strength of weak ties” is crucial for determining the extent of information dispersion in large-scale social institutions (Granovetter, 2005).

### Deliberative communication: An important variable in smallholders’ participation

Social structure and external environment could be the determining factors in smallholder’s participation in sustainable certification as both of the factors are proven to affect knowledge dissemination. However, smallholders’ participation could also be determined by rational choice. Despite these factors, other elements could be contributing to participative behaviour.

Communication is key to diffusion since it raises smallholders’ awareness and competence (Rogers and Shoemaker, 1971). However, communication should involve more than a one-way approach and should consist of a regular exchange of information that consists of questions and deliberation. It is applicable either between smallholders or between smallholders and an external agency (i.e., an extension officer). Sociologists define deliberative communication as “a cohesive set of more or less coherent understandings that defines the boundaries of thought and, consequently, behaviour” (Foucault, 2002). Deliberative communication is a specific manner of shaping relationships through language and other symbolic forms (Dragoi et al., 2011). Deliberative communication is, therefore, an essential form of communication in this situation.

Deliberative communication differs from traditional instructive communication, which works its way to the traditional “informs” or “instructs” mechanism. Smallholders were needed to perform specific actions or activities based on direction and order from the authority (Ahmad Rizal et al., 2021). Whereas in deliberative communication, smallholders can challenge or argue with any of the instructions based on their knowledge or experience. In agriculture practises, particularly in developing nations, it is typical for government agencies to push farmers/smallholders to obey orders without giving them the opportunity to speak (Friederichsen et al., 2013; Pincus et al., 2018). Deliberative communication thus introduces the concept of communicative power in which judgments made by smallholders are rational and it itself is a product of the “force of better arguments” (Flynn, 2004; Allen, 2012). A study reveals that smallholders exhibit greater adaptability and comprehension of a newly introduced innovation or intervention when they are permitted to actively participate in a dialogue with the instructor, who then actively reacts to their remarks (Pincus et al., 2018).

Thus, it is important to consider deliberative communication as an essential determinant in influencing smallholder’s participative behaviour to obtain sustainable certification.

### Responsible leadership

Responsible leadership is a leadership concept through the Habermasian Deliberative Democracy (Voegtlin, 2016). By definition, it is associated with awareness and consideration of the consequences of one’s actions for all stakeholders, as well as the exertion of influence by enabling the involvement of the affected stakeholders and by engaging in active stakeholder dialogue. Responsible leaders strive to weigh and balance the interests of the forwarded claims (Voegtlin, 2012; Ngah et al., 2022). In the case of smallholders and sustainable certification, a responsible leadership shall ensure all the relevant claims made by his or her followers either from the smallholders or other stakeholders are considered before making any decisions.

Responsible leadership has an important role during the discursive decision process. A leader shall be responsible not only for constructing the instrument to solve the problems but is also involved in the process. During the discursive decision process, a responsible leader would try to achieve consensus among the involved parties. This is achieved by weighing the arguments and balancing the interests of the stakeholders’ claims. This allows leaders to “*influence through cooperation and to aim for consensual solutions, as they interact not through a supervisor-subordinate relationship but eventually with equally powerful or resource commanding entities*” (Voegtlin et al., 2012, 4). Responsible leaders, thus, represent the position and the interest of their organization (e.g., smallholders or community groups) by joining the discourse with arguments that emphasize their point of view. This definition represents an ideal of responsible leadership that can encounter restrictions in the organizational process (Stansbury, 2009; Voegtlin, 2016).

Understanding the role of leaders in the current global setting can shed light on the impact of responsible leadership on the participation of smallholders in sustainable certification. Amid the globalisation, unpredictability, and interconnectedness of the business world, good leadership must try to reduce complexity and ambiguity among its followers. Today’s business leaders operate in a global, complex, uncertain, and interconnected world. Among the issues in this situation is the need to decrease complexity and uncertainty and establish a shared vision of the future. Responsible leadership also requires the prominent leader to show various levels of accountability in executive actions and decisions for their followers (Pless, 2007). Moreover, responsible leadership generates decisions based on followers’ communication and deliberation. As a result, every individual’s voice matters before any decision is reached (Voegtlin, 2016). Collective responsibility and disseminating ideas among followers are fundamental to responsible leadership.

However, the essential question here is how responsible leadership can mediate the relationship? The main reason is the difference of responsible leadership with other dyadic, leader-followers hierarchical thinking of leadership. As discussed above, the key aspect of responsible leadership is the ability to develop narratives based on the emphatic experience he or she experiences in the group. The characteristic will ensure the leaders have constant dialogue and deliberative communication with the group members, hence developing an understanding of their interests. Together with a good and sustaining relationship, leaders would be able to mobilize and align the energy of different people towards achieving common objectives (Howell and Avolio, 1992; Pless, 2007).

### Hypotheses and conceptual framework

**Figure 1:**
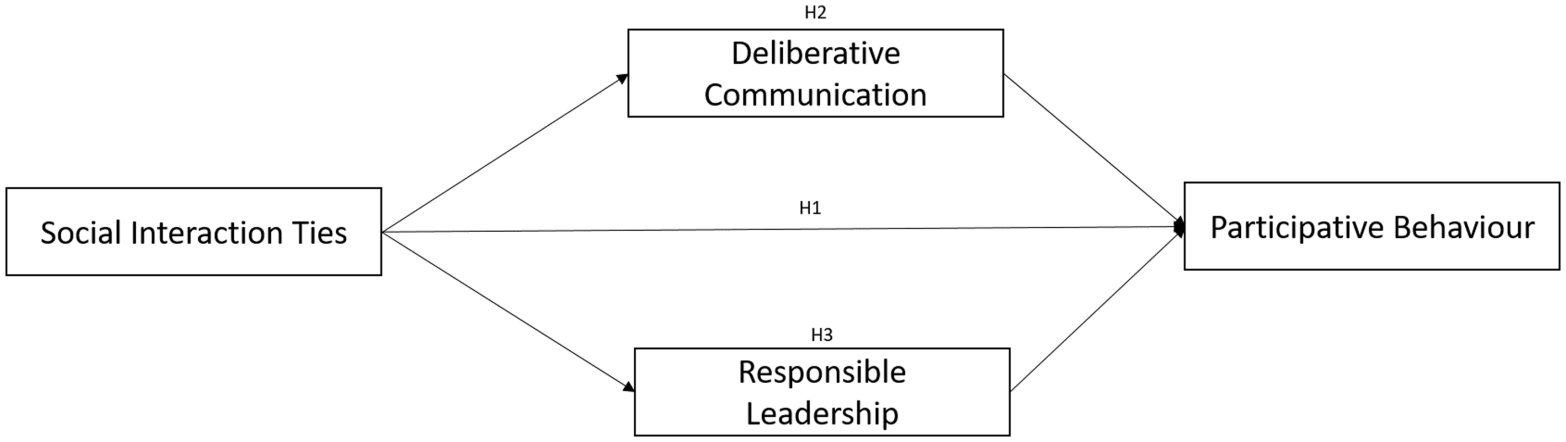
Conceptual Framework of the Study.

Based on the literature discussed, Figure 1 shows the conceptual framework of this study. There are 3 hypotheses developed for this study:

*H1*: Social interaction ties have a significant influence on smallholders’ participative behaviour

*H2*: Deliberative communication significantly mediates the relationship between social interaction ties with smallholders’ participative behaviour

*H3*: Responsible leadership significantly mediates the relationship between social interaction ties with smallholders’ participative behaviour

## Materials and methods

### Research design

This section describes the constructs used in this study. All the items in the constructs utilize a 5-point Likert scale as a measurement in the survey instrument. A quantitative approach was used in this study for the data collection and analyses. This shall enable the hypotheses testing that were set earlier in the development of the research conceptual framework. The target population and unit of analysis of the study were the palm oil smallholders in Malaysia who cultivate less than forty (40) hectares. Smallholders commonly refer to landowners who are given the right to plant oil palm in their respective areas which the area should not be more than 40 hectares (Kailany, 2011; Siduque, 2015). A set of survey was disseminated amongst the smallholder farmers in Malaysia. The survey itself was adapted and adopted from the previous studies. The detail of items used in the survey is explained in the measures section.

### Population and sampling

To determine the proper sampling technique, it is essential to identify the potential data analysis intended for this study. In this case, the most suitable analysis to determine the impact of constructs within an early exploratory model is Partial Linear Square–Structure Equation Modeling (PLS-SEM). Determination of sample size for this study is critical as there are cases where studies misused the advantages of PLS-SEM characteristics in analysing small sample sizes to produce the statistical output (Hair et al., 2019). The sample size in this is hence based on power analyses that consider the model structure, the anticipated significance level, and the expected effect sizes. These criteria are essential elements used by Hair and colleagues in developing their power tables known as the “minimum R-square method” to determine the proper sample size to be used in any PLS-SEM based study (Hair et al., 2017). This method, which builds on Cohen’s (1992) power tables for least squares regression, relies on a table listing minimum required sample sizes based on three elements (Kock and Hadaya, 2018). The first element of the minimum R-squared method is the maximum number of arrows pointing at a latent variable (i.e., construct) in a model. The second is the significance level used. The third is the minimum R^2^ in the model.

The required sample size was based on the minimum R-square method. Since this study has 3 arrows pointing toward the latent variable (dependent variable) and seeks 1% significance with a 0.10 minimum R - squared score, 176 samples are required (Hair et al., 2019). Despite that, to increase the confidence level of the findings, 440 samples were used in this study. This research employs random sampling as its sampling technique. Potential respondents were obtained from the local MPOB office. Random number generator software was used to generate random numbers. The respondents were selected based on their associated numbers in the list matching the numbers that appeared in the software. This approach shall minimise biases in selecting the respondents.

### Measures

#### Social interaction ties

Drawing from the study by Chiu et al. (2006), the constructs measure social relationships among smallholders. This includes their relationship in the community. The items used include, (1) I spend a lot of time interacting with members of my community, (2) I maintain close social relationships with members of my community, and (3) I have frequent engagement with members of my community.

#### Deliberative communication

A common communication method usually is one-directional involving orders from authority to the smallholders. This construct measures smallholders’ support for rational arguments. The measures were developed by Fast (2013) and contain five items: (1) I prefer to listen to both pro and against argument in my community before deciding which one need to be supported, (2) I prefer to consider different points of views from different members of the community in any discussion, (3) Disagreement are to be expected; I believe what matters is that we continue to cooperate in deciding discussion, (4) I prefer to support a discussion that embraces any suggestions from any participant, (5) Listening to other people’s view can broaden and enrich my views during the discussion.

#### Responsible leadership

This construct is based on instrument developed by Voegtlin (2012) and Maak et al. (2016). There are five items in the instrument: (1) I prefer leaders who demonstrate awareness of the relevant claims made by the smallholders, (2) I prefer leaders who consider the consequences of the decision for the affected smallholders, (3) I prefer leaders who include all the affected groups in the decision-making process, (4) I prefer leaders who weigh different smallholders claims before making a decision, (5) I prefer leaders who try to achieve a consensus among the affected smallholders.

#### Sustainability participation

Based on the instrument developed by Ni et al. (2016), this construct is to measure smallholder’s participation in CSPO. The items include: (1) I actively ensure that my plantation complies with the regulation underlined in CSPO (MSPO/RSPO), (2) I will ensure my plantation always possessed CSPO (MSPO/RSPO), and (3) I actively involved during the application of CSPO certification process (MSPO/RSPO).

### Data collection

This study was conducted in 2020 and 440 smallholders were involved. As stated earlier, the list of potential respondents was obtained from MPOB, Malaysia respected authority on the oil-palm commodity. Then, by using a random number generator, the respected respondent was identified. Respondents were then instructed to answer the developed questionnaire with the assistance of enumerators. The obtained data were then analysed using the PLS-SEM. To evaluate the robustness of the measurement model, reliability, convergent, and discriminant validity assessments are conducted. Then, this study’s structural model was evaluated for path coefficient, predictive power, significance, and effect size. Analyses were run on SMART-PLS 3.0.

#### Data reporting and analysis

Firstly, data collected from this study will be analysed for demographic findings. This study then will employ Partial Least Square-Structural Equation Modelling (PLS-SEM) for inferential statistics as consequently the hypothesis testing (Hair et al., 2014; Henseler et al., 2016). The inferential analysis by using PLS-SEM can only be conducted if the measurement model, as well as the structural model validity and reliability, were assessed. Both structural and measurement models used in this study passed the assessment. Table 1 shows the score of each assessment comprising of Cronbach Alpha score for reliability analysis, composite reliability and average variance extracted (AVE) for convergent validity analysis. Additionally, the discriminant validity of this model was also determined by it Heterotrait-Monotrait (HTMT) score where each construct managed to obtain a score less than 1 indicating high discriminant validity (Ramayah et al., 2018).

**Table 1:** Cronbach Alpha, composite reliability and average variance extraction (AVE) for each construct.

Construct	Composite reliability	Reliability (Cronbach Alpha score)	Average variance extraction (AVE)
Social Interaction Ties	0.812	0.862	0.591
Deliberative Communication	0.734	0.807	0.678
Responsible Leadership	0.781	0.812	0.737
Participative Behaviour	0.847	0.792	0.663

## Findings

### Demographics

More than half of the respondents are 46 years old and above. It shows that smallholder oil palm farming is an ageing society. Similarly, the majority of the respondents (54%) have more than 15 years of experience as oil palm smallholders. Despite the definition of smallholders as the farmer that operate less than 40 hectares of oil-palm plantation, the majority of respondents in this study which are 91% of total respondents operated plantation that is less than 3 hectares (3 hectares is equivalent to 7.4 acres) The details of demographic findings are shown in Table 2.

**Table 2:** Respondent profiles and demographics.

Respondent profiles	Frequency
*Respondents age*	
<25 years old	8
25–35 years old	82
36–45 years old	113
46–60 years old	182
>60 years old	55
*Education level*	
No formal education	10
Primary school	48
Secondary school	314
Post-secondary school (e.g., Technical certificate, diploma)	36
College degree (e.g., Bachelor’s Degree and above)	32
*Years as a smallholder*	
<5 years	36
6–15 years	166
16–25 years	192
>25 years	46
*Plantation size*	
<1 acre	102
1–3 acre	164
3–7 acre	136
>7 acre	38

### Multi-collinearity assessment

The collinearity issue is important in validating structural model integrity to avoid two sets of constructs to causally related (Ramayah et al., 2018). Collinearity is measured through variance inflation factor (VIF). A VIF value of 5 or higher indicates a potential collinearity problem (Henseler et al., 2014). All the inner VIF values of all sets of predictor constructs in the structural model are less than 5, indicating lateral multicollinearity is not a concern in the study and further examination of the model can be conducted.

### Significance and relevance of the structural model relationship

Investigating the significant level and t-statistics for all paths are important in order to test the developed hypotheses which measure the impact of the relationship between constructs. Table 3 shows all the path coefficient t and *p* values for each of the relationships. To obtain the t and p values for each relationship, bootstrapping analysis by using SmartPLS 3.0 were conducted. 5,000 sub-samples were generated and the confidence interval was measured by using “Bias-Corrected and Accelerated (BCa) with two-tailed test (Hair et al., 2017). Thus, a t-value of more than 1.96 with p values <0.05 indicate a significant relationship between the two constructs (Ramayah et al., 2018). The findings indicate that social interaction ties have a significant influence on smallholders’ participative behaviour. Thus, the first hypothesis of this study (H1) is accepted.

**Table 3:** Path coefficient and *p*-values for the study.

Relationship	Std beta	*p* values	Path coefficient significance
Social Interaction Ties → Participative Behavior	0.655	<0.001	Significance
Social Interaction Ties → Deliberative Communication	0.637	<0.001	Significance
Social Interaction Ties → Responsible Leadership	0.741	<0.001	Significance
Deliberative Communication → Participative Behavior	0.518	<0.001	Significance
Responsible Leadership → Participative Behavior	0.231	0.002	Significance

### Assessment of mediation analysis

The mediation models were tested to examine the indirect effects of deliberative communication and responsible leadership on the relationship between social interaction ties, shared identity and social norms on smallholder’s participative behaviour in the sustainable scheme. The bootstrapping analysis has shown that all two indirect effects are significant. There are:

- β1 = 0.329 (*t*-values = 3.615)
- β2 = 0.218 (*t*-values = 2.937)

The indirect effects 95% Boot CI Bias Corrected: β1 (LL = 0.152, UL = 0.512), β2 (LL = 0.072, UL = 0.363) show that each Upper Level (UL) and Lower Level (LL) of each relation do not straddle a 0 in between indicating there is a mediation in the relationship (Preacher and Hayes, 2012; Ramayah et al., 2018). Table 4 shows the detail of the mediation analysis in the study. Findings from these analyses indicate that the second (H2) and third (H3) hypothesis of this study is accepted.

**Table 4:** Mediation analysis of the study.

Mediating relationship	Std beta	*p* values (significance level)
Social Interaction Ties → Deliberative Communication → Participative Behavior (β1)	0.329	<0.001^**^
Social Interaction Ties → Responsible Leadership → Participative Behavior (β2)	0.218	0.003^**^

** indicate a significant *p*-value with the rejection value of *p* < 0.05.

## Discussion

### The significance of social structure in sustainable farming psychology

The findings demonstrate that social structure component which is social interaction ties have a substantial impact on smallholders’ participatory behaviour. The findings also indicated the importance of social capital, which is manifested through social interaction linkages. Smallholders collaborate closely and rely on one another for communication, information sharing, and support. In contrast to other regions of the world, primarily in the developed country, where a single individual farmer might manage dozens of hectares of land with the presence of mechanization, a plantation in Malaysia is rather operated modestly. Smallholder oil palm plantation managers often handle less than 4 hectares of land, with multiple studies indicating that the average size of a plantation is approximately that scale (Martin et al., 2015; Siduque et al., 2015; Ni et al., 2016). Social interactions have the capacity to enlarge the individual’s network structure and eliminate structural gaps (Burt, 2001; Lazega et al., 2012). This feature allows information to be broadly shared and enables smallholders to have a better understanding of the sustainable certification, which in turn leads to more participatory behaviour.

The importance of social interaction as a determining factor has been demonstrated in this study. Considering smallholders in Malaysia are observed to be closely connected, social interaction has an impact on their social structure. Sociological research has long emphasised the significance of interaction. Scholars have underlined the importance of social contact in shaping and influencing human decision-making (Jenkins, 2014; Saad et al., 2017). Mamat and colleagues found that the group’s development happened collaboratively in the community through a qualitative study conducted by them amongst oil palm smallholders in Pahang, Malaysia (Zufri Mamat et al., 2014). The community seeks to embrace modernization while maintaining its traditional values. This shift in cognitive viewpoint on identity among the members of the society has been demonstrated to be structural. In our study, the interaction’s effect on this situation is comparable with the past study. Interaction is a key factor in a person’s cognitive decision-making. Thus, in cases of sustainable certification, adopting the new policy is not totally up to a single person. This study found that living in a community created a feeling of shared identity, and a person’s grasp on community interaction affected his or her decision to pursue sustainable certification.

### Mediating capability of deliberative communication

This study indicated that deliberative communication mediates the relation between social interaction ties and participative behaviour. Deliberative communication refers to the concept of communicative power in which judgments made by smallholders are rational and it itself is a product of the “force of better arguments.” Thus, farmers of smallholders are not simply accepting any information communicate to them, they also are able to participate actively in the communication process. This include challenging the idea and having dialogue or discussion during the process. Findings from this study is also similarly reported in other studies. For example, in a study amongst farmers in Sub-Saharan Africa, the researchers found that constant communication that focuses on developing common grounds is essential to enhance the co-learning approach amongst farmers and educators (Marinus et al., 2021). Similarly, in a study conducted in Australia, the researchers reported that having an open communication structure between farm management and farmers is essential to improve productivity, product quality and profits (Klocker et al., 2020). Both findings reaffirm our findings on the mediating capability of deliberative communication amongst smallholders.

The working mechanism is explained by the process of social interaction between actors or in this case between smallholders and other actors (e.g., smallholders, extension officers and fruit dealers). The process leads to the exchange of ideas and also a clash of beliefs. According to Habermas, social interaction leads to communication and deliberative communication emphasizes ‘communicative rationality where the rational is an outcome of argument is pivotal. (Habermas, 2015). In the case of this study, deliberative communication shall first allow dual interaction between agencies involves. Hence, both of them can share and present their arguments. This ensures a non-one-way type of communication was implemented as the form of communication proven could be detrimental, especially to smallholders’ awareness of new policies and issues. Furthermore, deliberative communication was shown to lead to better knowledge construction (Gastil et al., 2008). Thus, it was proven in this study that deliberative communication leads to a positive impact on smallholders’ participative behaviour in sustainable certification.

### Mediating capability of responsible leadership

Finding from this study indicates that responsible leadership mediate the relationship between social interactions and smallholders’ participative behaviours. Responsible leadership focus on the ability of leaders to generate decisions based on followers’ communication and deliberation. As a result, every individual’s voice matters before any decision are reached (Voegtlin, 2016). The finding is consistent with other studies. In a study conducted amongst organic grain producers in the United States Corn Belt, the researchers identify that farmers are looking for leaders that are able to hear their concerns and suggestions in improving innovation dissemination in that area. They believe that this will strengthen the support mechanism that is much needed to propagate the process (Han et al., 2022). Furthermore, in the study amongst agro-forestry farmers, it is found that leaders who are able to create close connections with their followers will be able to enhance information sharing amongst the farmers. The information is crucial for any adoption of new methods or innovation (Lin et al., 2021).

Arguably, the mechanism of responsible leadership is related to the leaders’ cognitive ability to recognize, comprehend, and reflect their interests, needs, values, and demands in a connected, complex, integrated, and balanced manner with their followers (Stahl and Sully de Luque, 2014). This cognitive ability provides the leader’s resources in dealing with a wide spectrum of inquiries and attitudes of its followers. The increased amount of social interaction shall increase cognitive complexity, hence with sufficient resources, responsible leadership would be able to overcome the hindrance. Thus, leading its followers towards participation in sustainable certification. In the case of palm oil sustainable certification, not only do the leaders need to understand the behaviour and cognitive ability of their followers but there have also need to comprehend the complexity and essence of the certification itself. Failure to have that will result in an imbalance of understanding which contributed to the inability to convince his or her followers on the issues.

Furthermore, the development of responsible leadership is strongly rooted in emotional and moral experiences in the past which progressively develops from time to time (Kohlberg, 1981; Pless, 2007). The emotional and moral experience later shall develop into a sense of belonging as a product of social interaction. Hence, it is not surprising for social interaction and responsible leadership to be correlated. This is important as responsible leadership needs to understand the very fundamental struggle of its group. The situation was recorded in an empirical study conducted on paddy farmers in Perak. The farmers choose to believe their peers and colleagues for new information pertaining to innovation more than their belief in the extension officers despite the latter possess authority and better knowledge on the issue (Nordin et al., 2015). This is an example of cognitive complexity where the extension officers are unable to convey the message according to the farmers’ cognitive ability. Hence, it is why responsible leadership is shown to mediate the relation between social interaction ties and participative behaviour.

## Conclusion

The study examined the impact of social structure on smallholders’ decision-making to participate in sustainable certification schemes. This study was motivated by limitations found in both academic literature and industrial practices. As the most productive oil seed crop, oil palm is used for both edible oil and the bio-diesel market. However, sustainability has become an issue and initiative has been taken including the introduction of sustainable certification. Despite the rigorous approaches introduced to ensure smallholders are getting certified, the results are below expectations. The study was significant in identifying the underlying factors that influence smallholders’ decision to participate in the certification. Previous studies have emphasized the utility and economic dimensions while the findings from this study shed light from the social perspective taking into consideration social structure elements. Drawing on the social paradigm, this study argued that smallholders’ decision-making could be explained by social interaction. Moreover, this study has also identified the mediating impact of both deliberative communication and responsible leadership on the relation between social interaction and smallholders’ participative behaviour.

Thus, this study provides insights based on the scientific and validated approach to practitioners and scheme owners. It was proven in this study that social interaction, deliberative communication and responsible leadership lead to smallholders’ participation in sustainable schemes. Extension officers could initiate discussions and roundtable sessions between smallholders and other smallholders or between smallholders and officers. The aim of the session is to increase social interaction and deliberative communication between smallholders. Eventually, it enhances knowledge and awareness dissemination. It is also important for scheme owners to promote non-formal discussions and increase the volume of communication as of this study show communication and social interaction are essential.

Moreover, this study also shows to practitioners that responsible leadership mediated social structure constructs. This type of leadership should be promoted not only on smallholders – leaders’ relationship but also smallholders – extension officers’ relationship. Extension officer’s approach on smallholders is vital and it is not surprising the conventional top-down approach were consider normal practice especially involving government agencies (Redza et al., 2014). Hence, sustainability scheme owners such as MPOB could steer the leadership approach in their respective extension officers onto responsible leadership. This shall allow further enhancement in smallholders participative behaviour.

There were however several limitations in the study. A deeper analysis of social interaction between actors in the social structure could provide some insights into the structural holes that exist in social networks when there is a lack of direct contact or tie between two or more entities (Burt, 1992). It is important to reiterate that this study and several previous studies have shown that smallholders live in clusters. It means that smallholders interact with one another creating a bunch of clusters. The clusters may be connected by an actor known as ‘bridging ties’ (Lin et al., 2001). However, there might be cases where there are clusters that are isolated from other clusters. Hypothetically, the isolated clusters do not receive a similar impact of social interactions which may result in a lack of access to information. This phenomenon is called structural holes. The inability of this study to investigate the effect of structural holes on smallholders’ participative behaviour provides opportunities for further studies.

Future studies could possibly look on the moderating impact of structural holes. Furthermore, it could also identify which actors withing the group of smallholders has better influencing capabilities. Findings the individual capabilities shall strengthen findings of this study and further enhance the literature on social structure impact towards sustainability behaviour amongst farmers.

## Data availability statement

The raw data supporting the conclusions of this article will be made available by the authors, without undue reservation.

## Ethics statement

Ethical review and approval was not required for the study on human participants in accordance with the local legislation and institutional requirements. The patients/participants provided their written informed consent to participate in this study.

## Author contributions

SN and AR: conceptualization, methodology, and writing—original draft preparation. SN: resources and writing—review and editing. All authors contributed to the article and approved the submitted version.

## Conflict of interest

The authors declare that the research was conducted in the absence of any commercial or financial relationships that could be construed as a potential conflict of interest.

## Publisher’s note

All claims expressed in this article are solely those of the authors and do not necessarily represent those of their affiliated organizations, or those of the publisher, the editors and the reviewers. Any product that may be evaluated in this article, or claim that may be made by its manufacturer, is not guaranteed or endorsed by the publisher.
